# Exploring the Influence of a Smartphone App (Young with Diabetes) on Young People’s Self-Management: Qualitative Study

**DOI:** 10.2196/mhealth.8876

**Published:** 2018-02-28

**Authors:** Gitte Reventlov Husted, Janne Weis, Grete Teilmann, Pernille Castensøe-Seidenfaden

**Affiliations:** ^1^ Pediatric and Adolescent Department Nordsjællands Hospital University of Copenhagen Hillerød Denmark; ^2^ Department of Neonatology Rigshospitalet University of Copenhagen Copenhagen Denmark

**Keywords:** mHealth, diabetes mellitus, type 1, youth, self-management, qualitative research

## Abstract

**Background:**

Adequate self-management is the cornerstone of preventing type 1 diabetes mellitus (T1DM) complications. However, T1DM self-management is challenging for young people, who often struggle during the transition from childhood to adulthood. The mobile health (mHealth) app Young with Diabetes (YWD) was developed in collaboration with young people to enhance their T1DM self-management during this transition.

**Objective:**

The purpose of this study was to explore the influence of YWD on young people’s self-management during a 12-month period.

**Methods:**

A qualitative explorative approach was used, comprising a purposive sample of 20 young people (11 females and 9 males, ages 15 to 23 years, with app use of 3 to 64 days) from 3 pediatric and 3 adult departments. Participants were interviewed individually using a semistructured interview guide. Data were collected from January to March 2017 and analyzed using thematic analysis.

**Results:**

A total of 5 themes were identified: (1) not feeling alone anymore (“we are in this together”); (2) gaining competence by sharing experiences and practical knowledge (“they know what they are talking about”); (3) feeling safer (“it’s just a click away”); (4) breaking the ice by starting to share thoughts and feelings and asking for help (“it is an outstretched hand”); and (5) lack of motivating factors (“done with the app”). Young people reported that YWD promoted self-management by peer-to-peer social support, exchanging messages with health care providers, and sharing YWD with parents. Participants recommended YWD as a supplement to self-management for newly diagnosed young people with T1DM and suggested improvements in app content and functionality.

**Conclusions:**

The mHealth app YWD has the potential to support self-management. In particular, peer-to-peer support reduced feelings of loneliness and helped young people to gain knowledge and skills for managing T1DM. A need exists for alternative ways to train health care providers in using YWD and to support collaboration between young people and their parents to further improve young people’s self-management of T1DM.

## Introduction

### Background

Type 1 diabetes mellitus (T1DM) is a demanding disease for young people, who struggle to learn to self-manage their condition during the transition from childhood to adulthood [1,2]. As young people gradually become more independent, they are expected to take on responsibility for T1DM management that includes administering daily insulin, measuring blood sugars, and counting carbohydrates to meet the recommended glycemic control target [3]. However, new lifestyles and physical, cognitive, and social changes challenge daily T1DM self-management routines [4]. This often results in impaired glycemic control [5,6], increased risk of acute complications [7], and early onset of long-term complications [8,9]. In addition, young people with T1DM often skip clinical visits, endangering their current and future health [5]. Flexible engagement and continuity with health care providers and ongoing support from parents are still needed [10,11]. However, current routine care [5,6] does not seem to meet young people’s need for T1DM self-management support [12-14].

Mobile health (mHealth) apps seem to be suitable tools for engaging young people in self-management by providing information and optimizing interaction with health care providers and parents [15-19]. However, most mHealth studies to date have been conducted among adults. A review of mHealth apps identified 2 apps only that support T1DM self-management among adolescents [20]. Cafazzo et al showed an improvement in the frequency of blood glucose monitoring when testing an app to facilitate feedback on automated blood glucose readings [21], whereas Frøisland et al demonstrated an increased understanding of applied knowledge when testing the combination of a picture-based diabetes diary and a text messaging service [22]. Finally, a recent study by Holtz and colleagues reported that a patient-centered mHealth app for adolescents with T1DM and their parents seemed to enhance their collaboration [18]. In conclusion, the use of apps appears to have the potential to improve current patterns of providing self-management support [19]; however, there is a lack of evaluation studies focusing on the effect of mHealth apps on young people’s management of their long-term conditions.

### Young With Diabetes App

The Young with Diabetes (YWD) mHealth app was developed in a mixed-methods design based on a participatory and iterative approach [23] in collaboration with young people with T1DM, parents, health care providers, a team of health researchers, and information technology (IT) consultants. The goal of YWD was to provide a supplemental tool to support young people in developing T1DM self-management knowledge and skills during the transition from childhood to adulthood.

YWD is based on a family-centered approach [24] as recommended by transition guidelines [3,25,26] to improve self-management [1], defined as “an active, daily and flexible process in which youths and their parents share responsibility and decision-making for achieving disease control, health, and well-being through a range of illness-related activities” (p 92) [27]. Acquiring self-management in living with T1DM is a gradual process.

**Figure 1:**
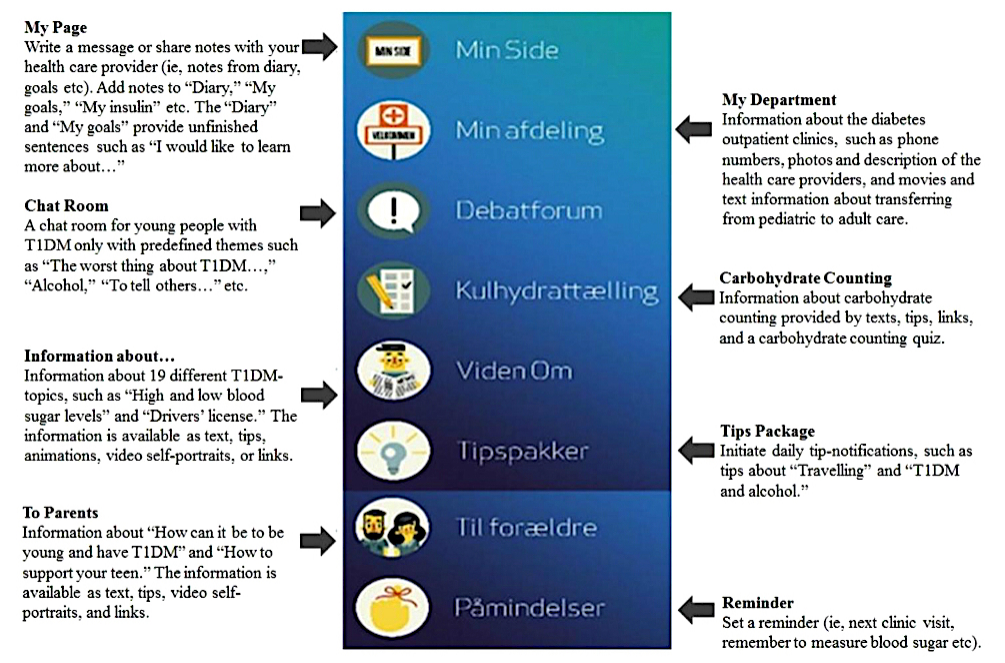
The eight main functions of the Young with Diabetes app. T1DM: type 1 diabetes mellitus.

YWD comprises 8 main functions (Figure 1): (1) My Page enables users to contact their health care provider and write notes, (2) My Department contains information about diabetes outpatient clinics, (3) Chat Room offers peer-to-peer interaction, (4) Carbohydrate Counting, (5) Information about T1DM, (6) Tips Package provides daily T1DM tips, (7) To Parents provides information for parents on how to support their teen, and (8) Reminder. Further details are provided elsewhere [23]. The YWD content did not change during the test period.

Young people, their parents, and health care providers received the same version of YWD, except the Chat Room that was only available for young people. Anonymity in the Chat Room was encouraged with the use of nicknames.

The aim of this study was to explore YWD’s influence on young people’s self-management during a 12-month period.

## Methods

### Design and Participants

A qualitative exploratory interview study was embedded in a 12-month randomized controlled trial (RCT) testing the effect of YWD on young people’s self-management skills. A purposive sample [28] of young people with T1DM who had completed the RCT study in the intervention group was recruited from January to the end of March 2017. They were invited to participate by the last author at the final data collection visit. 

Young people and their parents were randomized to the intervention group (YWD, n=76) or control group (n=75), and participants in the YWD group downloaded the app on their mobile phone or tablet. PC-S provided an initial 10 min introduction of the app to the participant in person or by telephone. Participants were encouraged to use YWD between clinical visits and in collaboration with parents and health care providers. No prompts or reminders triggered app use. All young people had a mobile phone.

Health care providers were encouraged to use the app in the outpatient clinic. YWD-trained diabetes team members, including physicians, nurses, and dieticians (n=39) with at least 1 year of diabetes outpatient clinic experience, provided the YWD intervention as part of usual practice. Monthly face-to-face sessions were offered to refresh the use of YWD, and a telephone hotline was available in case of technical issues.

### Data Collection

Individual interviews were conducted between January and March 2017 from 1 to 3 weeks after the RCT was completed. The interviews were conducted by GRH (an experienced female researcher who did not know the participants). GRH interviewed participants independently of their parents in their homes (n=19) or at school (n=1), using a semistructured interview guide (Multimedia Appendix 1) that included exploring individual reasons for variations in outcomes. The interview guide was inspired by an empowerment approach as defined by Anderson and Funnell [29]. The process of empowerment is defined as: “...the discovery and development of one’s inborn capacity to be responsible for one’s own life. People are empowered when they have enough knowledge to make rational decisions, control, resources to implement their decisions and experience to evaluate the effectiveness of their actions” (p 11) [29]. Interviews lasted for 35-60 min and were digitally recorded, transcribed verbatim, and checked for accuracy [30] by the first author. Transcripts were uploaded to NVivo software (QSR International version 11, QSR International Pty Ltd, Doncaster Victoria, Australia) to organize data and support the analysis.

Outcomes measures for the RCT included posttrial hemoglobin A_1c_ (HbA_1c_) levels (primary outcome) and scores on 3 psychometric scales (secondary outcome): Perceived Competence in Diabetes (PCD) [31], Health Care Climate Questionnaire (HCCQ) [31], and Problem Areas in Diabetes (PAID-20) [32]. These scores, along with baseline characteristics, were used to characterize interview participants.

The study was approved by the Danish Data Protection Agency (no. 04015 NOH-2015-031) and performed in accordance with the ethical recommendations of the Helsinki Declaration. Ethical approval of interview studies by Research Ethics Committee is not necessary in Denmark (no. 15000468). An information leaflet to participants stated that data would be treated confidentially and anonymously and that they could withdraw from the study without consequences for their treatment and care at the outpatient clinic. Written consent was obtained from all participants and from parents if participants were younger than 18 years. After each interview, GRH spent some time with participants to make sure they felt comfortable having shared their experiences with app use.

### Data Analysis

Data were analyzed using a 6-phase thematic analysis provided by Braun and Clarke [33]: (1) familiarization with the data, (2) generating initial codes, (3) interpreting and sorting codes into themes, (4) reviewing themes for coherent patterns, (5) deﬁning and naming the themes, and (6) producing the report [33]. GRH analyzed all interviews, generating initial codes and potential themes before discussing them with coauthors.

Subsequently, GRH and JW refined the categorization of codes into potential themes. Initially, this was an inductive process, followed by applying a more deductive approach during steps 3 and 4 to explore whether the app content and functions met young people’s needs [1,5]. Throughout all phases, constant checking of data extracts, codes, and themes against each other and the entire dataset was performed.

Credibility was addressed by researcher triangulation throughout the analysis, with GRH, PC-S, and GT having experience with T1DM and GRH, JW, and PC-S having experience with qualitative research. Feedback from the researchers was discussed at meetings until consensus was reached. Bias was diminished by having a coresearcher, who had not participated in the development of the app, to handle the analysis. Transferability was ensured by offering thick descriptions, dependability by providing quotes from informants, and conﬁrmability by thoroughly describing the processes of sampling, data collection, and analysis [34].

## Results

In total, 22 young people were invited to participate; 2 (1 female) declined due to exams or illness. The final sample comprised 20 young people (11 females and 9 males; age range, 15-23 years). They differed in app use and in the primary and secondary outcome measures (Tables 1 and 2).

Although young people still found daily self-care tasks difficult, YWD was experienced as a valuable tool to support T1DM self-care:

You damn need to do something [to self-manage T1DM]...but it [YWD] has helped me to do some of the daily work.

20-year-old female, ID17

This was identified through 5 themes: (1) not feeling alone anymore (“we are in this together”); (2) gaining competence by sharing experiences and practical knowledge (“they know what they are talking about”); (3) feeling safer (“it’s just a click away”); (4) breaking the ice by starting to share thoughts and feelings and asking for help (“it is an outstretched hand”); and (5) lack of motivating factors (“done with the app”). In the following section, the findings are detailed.

### Theme 1: Not Feeling Alone Living With T1DM Anymore—“We Are in This Together”

The Chat Room proved to be the most important function of YWD. Finding peers who faced the same challenges in T1DM self-management was enlightening for all interview participants; they felt that:

...we are in this together.

20-year-old female, ID1

Feelings of loneliness were relieved when young people became aware that their struggles were familiar issues among their peers. The possibility of contact with peers made their current challenges and worries easier to bear, as did knowing that they were not alone in having these burdens. Some participants had never met other young people living with T1DM, as illustrated here by a male:

I have gained a slightly better understanding, because before I had the app it was a little difficult to realize how others my age felt because there is no one at my school...that has diabetes...I think it is good to know that I am not the only one that has these problems or thinks about the same things, like the future with diabetes and all that.

18-year-old male, ID15

**Table 1:** Participant characteristics (n=20).

Characteristic		Value	
**Gender, n (%)**			
	Female		11 (55)
**Age in years, mean (SD, range)**			
	At baseline		18 (2.60, 14-22)
	At diagnosis with diabetes		9 (3.95, 2-16)
Diabetes duration at baseline in years, mean (SD, range)		9 (4.62, 3-18)	
**Insulin regimen, n (%)**			
	Multiple daily injections of insulin		11 (55)
	Pump		9 (45)
**Parental involvement, n (%)**			
	Participant lives with both parents		9 (45)
	Divorced		10 (50)
	At least 1 participating parent		13 (65)^a^
**Pediatric site, n (%)**			
	Pediatric and Adolescent Department, Nordsjællands Hospital, Hillerød		4 (20)
	Pediatric and Adolescent Department, Herlev		5 (25)
	Pediatric Department, Roskilde		1 (5)
**Adult site, n (%)**			
	Department of Cardiology, Nephrology and Endocrinology, Nordsjællands Hospital, Hillerød		2 (10)
	Steno Diabetes Center		6 (30)
	Department of Endocrinology, Køge		2 (10)
Transfer to adult care, n (%)		2 (10)	
Active app days, mean (SD, range)		19 (15.87, 3-64)	

^a^Mother (n=8), father (n=1), both mother and father (n=4).

**Table 2:** Participant scores at baseline and at the end of the 12-month trial (n=20).

Outcome measures	Range of possible scores	Baseline, mean (SD)	12 months, mean (SD)
HbA_1c_^a^	-	83 (20)	82 (19)
PCD^b^	5-35	27 (8)	28 (7)
PAID^c^	0-100	27 (21)	28 (19)
HCCQ^d^	5-35	28 (7)	29 (6)

^a^HbA_1c_: hemoglobin A_1c_, mmol/mol; assesses blood sugar control.

^b^PCD: Perceived Competence in Diabetes; assesses patients’ experience of feeling able to successfully manage diabetes; higher scores represent greater perceived competence.

^c^PAID: Problem Areas in Diabetes; assesses diabetes-related distress; higher scores indicate greater emotional distress, and a score ≥30 indicates elevated distress.

^d^HCCQ: Health Care Climate Questionnaire; assesses the degree to which patients perceived their health care providers as supporting their autonomy; higher scores indicate a high level of perceived support for autonomy.

The possibility of participating in chats or simply observing chat comments helped young people acknowledge that it was not always easy to self-manage T1DM. They realized that peers were familiar with their concerns, frustrations, and challenges, which helped reduce feelings of loneliness, as expressed by a female:

[I]t is good to know that you are not the only one with it...of course you have the support from your family and your friends, who say that you will get through it...but hearing it from someone who also has it and knows what you are going through and...to have others you can talk to, that is very, very nice.

18-year-old female, ID14

Feeling like everyone else and “being normal” (16-year-old female, ID9) occurred when young people were in the Chat Room. By reflecting on their peers’ ways of living, young people experienced normalization of T1DM into everyday life. This contrasted with the feeling of being different that often arose when spending time with friends without T1DM:

[O]ften you feel that you are walking around in your own little world, because you are surrounded by people that don’t have diabetes.

18-year-old female, ID14

The Chat Room provided a safe, closed space where a spirit of companionship arose even though the young people did not know each other. A female described the experience:

[O]ften when you are at school, you are reminded that you have diabetes...But when you are in that diabetes chat room, then you are among others, and it is like the diabetes things are something that you have in common with the others.

18-year-old female, ID14

### Theme 2: Gaining Competence by Sharing Experiences and Practical Knowledge—“They Know What They Are Talking About”

Young people shared T1DM experiences in the Chat Room, which helped them gain new knowledge and skills for managing their disease *.* They became aware of the difference between advice and information they received at outpatient clinic visits and the insights and real-life knowledge they received in the Chat Room:

...they know what they are talking about.

22-year-old male, ID3

They experienced health care providers as being unable to provide the person-specific information they sought. On the contrary, health care providers often discussed T1DM in general ways, for example, by looking at blood sugar curves trying to figure out the cause of fluctuations. The young people found that sharing experiences with peers provided valuable and reliable person-specific information, such as how to manage hypoglycemia or deal with challenges when traveling. This knowledge improved their self-management competencies, as described by a male:

[O]ne way is that a physician tells you how to do it, another thing is when the ones that have [T1DM], tell what they do to make their blood sugar levels drop or what they do when they travel, so that has helped me quite a lot.

18-year-old male, ID15

Young people became confident about trying out new ways of handling T1DM. They exchanged tips and tricks, such as precautions for driving, how to regulate blood sugar in relation to sports, and how to use the different functions of the insulin pump around meals to avoid fluctuating blood sugars.

There are people who have solutions to problems that you might not be able to find yourself.

16-year-old male, ID13

Easy access to the Chat Room provided young people with opportunities to immediately change self-care practices. As a female described:

There was one person who wrote that she used the basal [rate] in the pump...and I didn’t know that you could turn up the basal, then she wrote how to do it...that has helped me a lot, now it [the blood sugar level] doesn’t fluctuate as much.

19-year-old female, ID6

Young people gained practical knowledge that supported their participation in social life, such as going out with friends, while still feeling confident about taking care of their T1DM. 

Exchanging experiences in the Chat Room provided them with real-life scenarios of risky situations, such as drinking alcohol. They did not receive this type of information from their health care providers. From their peers, they received concrete instructions about how to act and cope, as described by a female:

...it has also helped me a lot with what to do if things go wrong when I drink and am at a party...

18-year-old female, ID14

Most young people had not used the Information about...section, often because they felt they knew everything about T1DM after having been diagnosed years previously:

Maybe sometimes it is just the words, “Information About,” where I think I don’t need to read that.

20-year-old female, ID17

Therefore, they did not consider the informational part of the app as an option for obtaining advice about how to self-manage different situations in real-life contexts. However, they were convinced that the information function of YWD would be very useful for young people newly diagnosed with T1DM. As a male put it:

...The category “Information About” would be real smart for new diabetics who don’t have any idea as to how the different things affect your body.

17-year-old male, ID18

### Theme 3: Feeling Safer Having the App—“It’s Just a Click Away”

Young people felt safer living with T1DM knowing that all the information they needed about their disease was available in YWD. This gave them a sense of freedom and peace:

It has been a relief for me that all my diabetes things are gathered in one location.

18-year-old female, ID14

Before using the app, they had often had difficulty maintaining an overall perspective on their T1DM while taking care of school, work, and youth life. Ready access to information and knowledge made self-management easier:

It’s just a click away.

20-year-old female, ID5

For instance, some young people used links in the app to figure out how to count carbohydrates, some gained new perspectives by watching video self-portraits, and some reached out to peers or health care providers *.* YWD functioned as a kind of “back up” (16-year-old male, ID13) in almost all aspects of life with T1DM from minor concerns to acute issues. Young people viewed the app as a lifeline and felt safe just having it, as described by a female:

...then it is more reassuring with the app...let us say you have a situation, and then you can quickly...sit and look it up.

20-year-old female, ID1

A few young people benefited from writing personal notes in the My Page function to organize their life with T1DM. Before using the app, they had expended a great deal of energy figuring out how their blood sugar responded to activities and food intake. By making notes about their reactions during different activities and in various circumstances, they gradually developed a personal guide to rely on in similar situations. This app function provided a *parking lot* for speculation and worry, helping ease their minds. As a female explained:

I have gotten a better perspective on it, so I don’t have so many different thoughts...and I have gotten it all gathered so I can access it directly and see, that is what I did the last time and create my own guides to what I need to do, so that has helped me a lot.

18-year-old female, ID14

Young people appreciated that the app’s informational sections were easy to understand and focused on the most important aspects of T1DM. They had greater confidence in the information presented by YWD than in information they had previously located online. This gave them a sense of safety, as expressed by a male:

Information about...,” that is professionals that have written that, it is not someone off some Google home page...so that makes it a lot more reassuring to have this app.

17-year-old male, ID13

### Theme 4: Breaking the Ice by Starting to Share Thoughts and Feelings and Asking for Help—“It Is an Outstretched Hand”

For some young people, YWD became a way to break the ice with their health care providers and parents. It supported them in sharing thoughts and feelings about their challenges of living with T1DM and asking for help.

Young people found that contacting health care providers through YWD was very informal, which facilitated writing messages:

I communicate with my nurse in a completely different way—I can be much more honest.

20-year-old female, ID17

Young people primarily wrote about practical things such as scheduling appointments or new prescriptions. They emphasized that health care providers did not introduce YWD during outpatient clinic visits or only referred to it superficially, such as by asking whether they had used the app. Young people had the impression that the app was not meant for collaboration. However, YWD made a profound difference in the few instances in which young people used it in collaboration with health care providers. For example, they used it to ask for help to lose weight or help to manage an eating disorder, topics that had not been discussed during clinic visits. A female described it this way:

I have had difficulties with my eating disorder, and that has not been something I have told my practitioners, so the app gave me an opportunity...to write them, because it was hard for me to say, either over the phone or face-to-face.

23-year-old female, ID10

Other young people used the “Unfinished sentences” in the My Page function as a way to break the ice and address unspoken difficulties in living with T1DM. They felt that doing so made it possible to talk openly about their thoughts and feelings:

The thing that I am worst at, regarding my diabetes...” that is a very accurate sentence, because it is like taboo.

20-year-old female, ID17

By sharing their thoughts and feelings with health care providers, young people suddenly experienced more continuity in those relationships. This promoted sharing successes and failures and receiving ongoing support, which not had been possible before.

It motivated young people to improve their self-management because health care providers immediately responded to their actions. A female explained it this way:

The thing about how many blood sugar levels I have measured in so and so many days, that I would never have told her, but she sees the progress and tells me about it and praises me for it and that would not have happened without the app.

20-year-old female, ID17

YWD also became a way for young people to break the ice and start to share thoughts and feelings about the challenges of living with T1DM with their parents and to ask for help when needed. Some young people and their parents looked at the video self-portraits or at posts in the Chat Room, competed in the Carbohydrate Counting Quiz, or looked at the information sections separately or together. They then began to talk about topics that previously had been difficult to discuss openly, as in the example of a male who had talked about sex with his father for the first time while looking at the app together:

[M]e and my dad, we talked about the issue of having sex with diabetes...that is probably not something I would have thought about normally—if there is a difference there...so because of the app we actually talked about some stuff.

17-year-old male, ID18

Young people described their parents as gaining a deeper and more nuanced perspective on the difficulties they faced in trying to self-manage T1DM. This changed the way parents and young people interacted about their self-management. Young people described their parents as seeming more eager to provide appropriate autonomy support, rather than admonishing or comforting them. As a girl describes:

We talk about it in a different way, because they are informed about it, and they have asked... “Have you had it like that...?” and I have said “I think, like all the others, that it is a shitty thing to have” ...then they have said: “If there is something we can do [as opposed to everything will be all right], we would like to help.” They somehow better understand how it works in our minds...how we feel about our diabetes.

18-year-old female, ID14

Young people felt that their parents started to show greater confidence in them, and they had the impression that their parents suddenly took their situation and frustrations more seriously. In some cases, this led to changes in their parents’ point of view, including relaxing rules as they became more confident that the young person could handle, for instance, alcohol. A boy stated the following:

Here in the beginning of last year, my parents wouldn’t allow me to drink, and now I have just gotten permission since we looked at the app together, there was a chat concerning alcohol and that has helped us a lot.

15-year-old male, ID20

### Theme 5: Lack of Motivating Factors—“Done With the App”

Despite the fact that young people appreciated YWD, some also expressed being “done with the app” after the first few months because no new information, quizzes, or video self-portraits were added during the trial. A male said:

It would be nice if it [YWD] was updated...it’s like Wikipedia, when you have read it, then you have read it, new knowledge won’t suddenly appear.

17-year-old male, ID18

Moreover, they noticed decreased activity in the Chat Room, which reduced their motivation for keeping the app. A male described the lack of motivating factors:

There aren’t enough who write, so it is kind of like...it is hard to see where the app is supposed to take us, when there aren’t enough who use it to answer and write.

22-year-old male, ID3

However, young people wanted to keep YWD, except for 3 male participants, aged 20 to 22 years, who no longer wanted it due to the static content. All participants had many suggestions for improvements to content and functionality to ensure more activity and make YWD more useful (Table 3).

**Table 3:** Suggestions for improving Young with Diabetes content and functionality. T1DM: type 1 diabetes mellitus.

Main function	Suggestions for improvements
My Page	Share the notes, diary, and messages with parents
	Visualize blood glucose readings in a graph and compare the readings with recommendations
	Set a blood glucose level goal and receive motivating feedback notifications
	Access your medical record, such as information about treatment and hemoglobin A_1c_
My Department	Automatic information from the hospital database about your health care provider, department, and phone number
Chat Room	Add a parent chat room
	Add closed chat rooms
	Add invitations to events
	Create your own chat themes and change the order of the predefined themes
	Include the opportunity to chat with a diabetes physician
	Create a profile similar to Instagram
Carbohydrate Counting	Include specific amount of carbohydrates
Information about T1DM	Continuously updated information, such as the newest research in T1DM Detailed information about treatment options, such as photos of devices
	Include quizzes on T1DM topics with varying levels. Upload new quizzes regularly
	Add an introduction to the video self-portraits and place the video self-portraits in front with a more obvious play button
	Improve the overview and shortcuts to the most frequently used T1DM apps
To Parents	Include information about what it is like to be a parent of a young person with T1DM
Other suggestions	Quick introduction to Young with Diabetes by a short animation describing content and functionality
	Customization, such as choosing the background, the start page, and placing the favorite sections in front
	Improve the intuitive interface by adding more visual icons and small information boxes
	Reduce scrolling of long text sections and reduce the number of “clicks.” Add shortcut options
	Add a narrator button to read the text for people with reading difficulties
	Fingerprint login

## Discussion

### Principal Findings

This qualitative study provides insight into the mHealth app YWD as a motivating factor and a supplemental tool to self-manage T1DM. In particular, young people experienced interactions in the Chat Room, exchanging messages with health care providers, and looking at the YWD with parents as useful features. Our findings show the importance of helping young people communicate with like-minded peers to share feelings, practical knowledge, and experiences. In addition, the findings indicate that YWD is a promising supportive tool to change communication patterns between young people and health care providers during and between outpatient clinic visits and between young people and their parents. Finally, YWD could be a tool for health care providers to address sensitive topics in outpatient clinic visits. However, improvements are also needed to maintain young people’s motivation for self-management.

### Comparison With Prior Studies

The Chat Room was found to be the most important part of the app, providing an online community for young people struggling with the same feelings, thoughts, and practical issues related to T1DM self-management. Young people felt they received support for self-managing specific diabetes situations and experienced diminished feelings of loneliness. Previous research has identified this kind of interaction as diabetes-specific social support [35], which is associated with reduced loneliness in living with T1DM [36]. Social support organized by health care providers as a kind of peer-to-peer support [37] has proven effective in preventing loneliness [38]. However, the literature on diabetes-specific peer support addresses a wide range of peer interactions and interventions [39,40], and the evidence is insufficient to determine the types of peer interactions, elements, and interventions most applicable to young people with T1DM [39]. YWD seems to have the potential to reduce loneliness, as compared with traditional outpatient clinical visits. Our findings emphasize that young people need help to connect with peers to share their experiences in a way that they cannot with parents, health care providers, and other social networks, as identified by Mayer et al [41].

In addition, untreated loneliness is known to contribute to diabetes distress [36,42]. Diabetes distress is high among young people emerging into adulthood [43]. In our study, young people experienced a slight increase in diabetes distress (PAID-20) scores (Table 2). The lack of effect on diabetes distress in our study could be explained by decreasing activity in the Chat Room during the trial period. Another potential explanation is that YWD alone does not provide sufficient support for young people with T1DM. Finally, it could be a result of the researchers bringing attention to the feelings of distress, supporting participants in identifying and reporting these feelings.

It is well known that health care providers play a significant role in supporting young people’s self-management [44]. However, not all health care providers feel confident using mHealth apps [45], and some may feel uncomfortable engaging with young people through technology [46,47]. This may explain why some young people perceived little interest from their health care providers in using the app collaboratively. Overall, YWD could not overcome barriers to frank discussions about sensitive topics, such as sex and alcohol, with which young people often struggle [38]. These topics should be addressed regularly in clinic visits [1]. However, the slight increase in HCCQ scores (Table 2) indicate that YWD may have the potential to complement health care providers’ traditional self-management support when used as an ongoing autonomous support in collaboration with the young people. In addition, YWD was able to slightly increase young people’s perceived competences in diabetes self-management identified by the PCD scores (Table 2).

Health care providers’ training in how to use YWD was very brief, lasting a single hour. This may not have been sufficient to help them feel confident in using the app in collaboration with young people and their parents [23]. Adaption and adoption of new technology require serious implementation work, including developing health care providers’ competence at performing new tasks and using the technology as intended [48]. Our study demonstrates that a critical need exists for guidelines on how to optimally train health care providers to use self-management apps as currently recommended [3,25,26].

Interestingly, we found that YWD use often changed interaction patterns between young people and their parents. The app created a platform for young people and parents to approach each other in a more constructive way and enable them to talk about sensitive topics by sharing video self-portraits or posts in the Chat Room or looking at the To Parents section. Parents often face challenges when trying to support their child in achieving self-management [18,49]. New ways of facilitating interactions that engage parents and young people during the transition from childhood to adulthood are needed [50,51]. YWD seems to have the potential to complement this process, but methods for encouraging collaborative use of mHealth apps by young people and their parents are needed; only 13 of 20 young people used YWD with their parents (Table 1). However, our findings suggest that combining an online space for exchanging peer support with support from health care providers and parents seems to facilitate more successful self-management, as also identified by Kowitt et al [52].

### Implications

The findings highlight the importance of addressing communication about illness in any patients with long-term conditions by focusing on improving peer-to-peer support as well as supporting the digital communication with health care providers. However, there is a need to focus on health care providers’ competence at using mHealth apps. Given the many available mHealth apps, it may be worthwhile to introduce a course during health education programs, such as in medical and nursing schools, focusing on how to use technology collaboratively with patients. This is supported by a recent study [53] in which medical students reported improved understanding of the involved issues and procedures and greater confidence in conducting a telehealth consultation after a course in telehealth skills [53].

In addition, YWD may be a valuable tool for people with newly diagnosed T1DM as recommended by participants in the study. The informational functions of the app may be most useful at an early stage of the disease. We intend to incorporate young people’s feedback and suggestions for improvements in content and functionality (Table 3) before testing YWD in a group of participants with newly diagnosed T1DM. Because the age at diagnosis of T1DM may be different compared with participants’ age in this study, further revision would be of interest to meet the needs of younger participants.

YWD may have the potential to provide a generic model for supporting young people with other chronic conditions and their parents and helping health care providers to engage with young people in ways that provide ongoing support. Despite disease-specific differences, young people with chronic conditions and their parents share many commonalities [16].

### Strengths and Limitations

One of the strengths of our study is the recruitment of participants with a variety of app use, age, gender, T1DM duration, affiliation with diabetes departments, and outcome measures. We consider this population as representative of the entire study group. In addition, patient-centered methods were used to assess YWD. Furthermore, the study used a rigorous qualitative methodology (thematic analysis), as described by Braun and Clarke [33], to provide rich and detailed data, rendering the freedom of combining an inductive and a deductive analysis to identify patterns within data. A limitation is the short-term nature of the study (app use range 3-64 days) and the varying levels of health care provider collaboration. Furthermore, we did not report parents’ and health care providers’ perspectives on using YWD and its impact on young people’s self-management. Currently, parents’ and health care providers’ perspectives are being explored in questionnaires and focus group interviews, respectively, to make a more informed case about the implications of YWD on all groups of users.

### Conclusions

The YWD app appeared to be a motivating factor in young people’s self-management of T1DM. In particular, peer-to-peer support, exchanging messages with health care providers, and looking at the YWD with parents were useful in supporting self-management. YWD changed communication patterns between young people, health care providers, and parents and provided young people with ongoing support. YWD is not effective as a stand-alone intervention, but it seems to have the potential to help young people, parents, and health care providers optimize T1DM self-management. A need exists to refine YWD according to users’ suggestions, to optimize and standardize health care providers’ use of YWD, and to further investigate how the app can be used collaboratively by young people, their parents, and health care providers.

## Appendix

Multimedia Appendix 1 Interview guide.

## Abbreviations

HbA_1c_: hemoglobin A_1c_
HCCQ: Health Care Climate Questionnaire
IT: information technology
PAID-20: Problem Areas in Diabetes
PCD: Perceived Competence in Diabetes
mHealth: mobile health
RCT: randomized controlled trial
T1DM: type 1 diabetes mellitus
YWD: Young with Diabetes
