# Supplementary material for: Exploring the Influence of a Smartphone App (Young with Diabetes) on Young People’s Self-Management: Qualitative Study
Source: JMIR Mhealth Uhealth. 2018 Feb 28;6(2):e43. doi: 10.2196/mhealth.8876 (PMC5856925; doi:10.2196/mhealth.8876)
Supplement: Multimedia Appendix 1 [file mhealth_v6i2e43_app1.pdf]

Multimedia appendix 1. Interview guide

TOPIC	MAIN QUESTION	SUPPORTING QUESTIONS
**Introduction**	Presentation<br><br>Purpose of the interview<br><br>Time frame	There are no right or wrong answers<br><br>Questions?
**Opening question**	Think about your participation in the app project – what have you gained over all by participating in the project?<br><br>In the questionnaire, in regard to the question: Has the app helped you? You answered Yes/No at 2, 7 and 12 months. Please, tell me why you answered Yes/No?	Can you give an example of how the app has helped you?<br><br>Can you give an example of how it was not able to help you?<br><br>Can you describe a specific situation where you needed to get concrete information on how to handle a situation but where you could not find help in the app (job/school/sport/party/hypo/food and carbohydrates etc.)?
**Use of YWD[a] in general** (Looks at the app with the participant)	Which parts of the app have you especially used?<br><br>Which parts of the app have been most important to you?<br><br>Which parts of the app have been of less importance to you?	Can you give an example of what you used it for?<br><br>What should have been different (function/content) in YWD so it had been more valuable for you?
**Support to feel like other young people** (Looks at the app with the participant)	In the questionnaire you wrote that the best part of the app was the …*Chat Room*. Can you tell a little bit more about that?<br><br>What is your experience with the video self-portraits?	Can you give an example?
**Use of YWD between outpatient clinic visits** (Based on the participant's baseline and final PCD scores)	Before you got the YWD app, what did you do if you faced a situation with your diabetes where you didn't know what to do and you needed help (e.g. fever, parties, travelling, alcohol, sex, sport)?<br><br>The intention behind the app was also to ease the contact with your physician/nurse/dietician between your outpatient clinic visits – e.g. write messages to them. How much have you used this function?	Can you give an example?<br><br>What has the app not been able to help you with between outpatient clinic visits?<br><br>What is the reason that you have/have not used this function?<br><br>Can you give an example of what you have written to your physician/nurse/dietician?
**Use of YWD at outpatient clinic**	How have you used the app during outpatient clinic visits with your	Can you give an example of how you

**visits** (Based on baseline and final HCCQ scores)	physician/nurse/dietician?<br><br>If the app should become a natural part of your outpatient clinic visits in future – what would that take?	have used the app during the visits?<br><br>Think about how your visits were before you got YWD – what has been the greatest difference, if there has been a difference?<br><br>Can you give an example?
**Handling anxiety for hypoglycemia** (based on baseline and final PAID-9 scores)	One of the questions in the questionnaire addresses worries about low blood sugars. I can see that you initially answered that it was (i.e…. not a problem) and at the end of the project you answered that it was (i.e....a minor problem) which indicates that you now experience that you are (i.e….a little worried about getting low blood sugars). What do you think is the reason that you have become a little worried about low blood sugars?	What impact has the app had on your fear of hypoglycemia?<br><br>Can you give an example of how YWD has made you a little worried?<br><br>What should be different in YWD to help you to be less worried about low blood sugars?
**General challenges living with T1DM (**based on baseline to final PAID scores)	When I see your answers to the questionnaire it looks as if you experienced (i.e….having more difficulty handling your everyday life before you got the YWD app). What do you think is the reason why it has become a little bit easier to handle your everyday life with diabetes?	What impact has the app had?<br><br>Can you give an example of how the app has helped you/not helped you?<br><br>What should be different in the YWD app to be more helpful in your daily life?<br><br>What other reasons could have had an impact on your challenges?
**Parents' use of YWD in collaboration with the young person**	Your mum/dad has participated in the project and has had the YWD app. How do you experience that your mum/dad has used the app?<br><br>What significance has it had for you and your daily life with diabetes that your mum/dad has had the app?	Can you give an example?<br><br>If you have used it in collaboration with your mum/dad – what have you used it for?<br><br>What would it take to use YWD more in collaboration with your mum/dad?<br><br>How have you noticed that your mum/dad has had the app?<br><br>Can you give an example?
**On their own feet and motivation for self-management of diabetes**	Whether the YWD app has helped the young participants in the project has also been assessed by measuring long-term blood sugar levels during the project period. How do you think your long-term test has been affected?<br><br>Are you interested in getting the results	Your long-term blood sugar level has decreased/increased from XX to XX during the project – what do you think is the reason for this decrease/increase?<br><br>What impact has YWD had on this decrease/increase?

	from me?	Can you give an example?<br><br>What should be different in YWD to help you obtain your goal of the long-term blood sugar level?
**Transfer from pediatric to adult department**	How have you used the YWD app in relation to your transfer from pediatric to the adult care diabetes department?	Which significance has it had for you that these departments were described in YWD?<br><br>Can you give an example?<br><br>Is there anything you have missed in YWD in relation to your transfer?
**Information on *My Department***	How have you used the app in relation to seeking information about your outpatient clinic?	Can you give an example of how you have used YWD in relation to your outpatient clinic?<br><br>What significance has it had to you that there were these parts in the app?<br><br>What do you miss in the app in relation to your outpatient clinic?<br><br>Can you give an example?
**Closing**	If you could give any suggestions on how to change YWD to be more helpful so you are able to manage daily life with diabetes even better – what would that be?	What has been the best/worst thing about YWD?<br><br>If a young person with diabetes contacted you and told that he/she had been offered the YWD app but was in doubt about what to use the app for, and he/she would like your advice concerning the app – what would you tell him/her?<br><br>We have talked about…..and I have noticed that you especially have liked xxx because xxx.<br><br>Is there anything we have not talked about, that you think is important to improve the YWD app in order to best help young people with diabetes to manage daily life with diabetes in future?

[a]YWD: Young with Diabetes
